# Standoff detection of bacterial spores by field deployable coherent Raman spectroscopy

**DOI:** 10.1038/s41598-023-29589-7

**Published:** 2023-02-14

**Authors:** Nicola Coluccelli, Gianluca Galzerano, Paolo Laporta, Kelly Curtis, Claire L. Lonsdale, Debbie Padgen, Christopher R. Howle, Giulio Cerullo

**Affiliations:** 1 Dipartimento di Fisica-Politecnico di Milano, Piazza Leonardo da Vinci 32, 20133 Milan, Italy; 2 Istituto di Fotonica e Nanotecnologie-CNR, Piazza Leonardo da Vinci 32, 20133 Milan, Italy; 3 Defence Science and Technology Laboratory, Porton Down, Salisbury, UK

**Keywords:** Raman spectroscopy, Ultrafast lasers, Nonlinear optics

## Abstract

Vibrational spectroscopies offer great potential for standoff detection of chemical and biological warfare agents, avoiding contamination to the operator and equipment. Among them, particularly promising is Coherent anti-Stokes Raman scattering (CARS) spectroscopy, using synchronized pump/Stokes laser pulses to set up a vibrational coherence of target molecules at a laser focus, which is read by further interaction with a probe pulse, resulting in the emission of a coherent beam detectable at a distance. CARS has previously demonstrated the capability to detect bacterial spores based on the Raman spectrum of the characteristic molecule calcium dipicolinate (CaDPA); however, a complex and bulky laser technology, which is only suitable for a laboratory environment, was employed. Here we develop a broadband CARS setup based on a compact, industrial grade ytterbium laser system. We demonstrate high signal-to-noise ratio detection of *Bacillus atrophaeus* spores at a concentration of 10^5^ cfu/mm^2^, at a standoff distance of 1 m, and an acquisition time of 1 s. Our system, which combines chemical specificity and sensitivity along with improved ruggedness and portability, paves the way to a new generation of instruments for real-world standoff detection of chemical and biological threats.

## Introduction

Standoff detection of chemical and biological warfare agents is a topic of growing interest for both the military and homeland security. Optical techniques offer a great promise in this respect, since they are intrinsically non-contact, avoiding potential contamination to the operator and equipment; however, reaching the required sensitivity and specificity poses a difficult detection problem. Laser induced fluorescence with ultraviolet excitation (UV-LIF) is a widely used technique, which can yield high signal intensities provided that energetic laser pulses are used for excitation^1,2^; however, it is rather unspecific, and the discrimination of biological agents may be challenging because frequently their fluorescence spectra are featureless and similar to those of other organic materials occurring in the environment^3^. Laser induced breakdown spectroscopy (LIBS) is another detection method that uses high energy laser pulses to break down the sample into a plasma, which in turn emits light at characteristic frequencies which allows for determination of the elemental composition (ions, atoms, and molecular fragments). Beyond the questions of LIBS signal strength and sample damage inherent to the technique, it must be noted that biological agents display significant variability in their elemental content, depending on the way in which they were grown or handled, which limits the specificity of LIBS and narrows the scope of its applicability in biological-agent detection^4,5^. Photoacoustic spectrosocpy is an emerging technique which has shown some potential for detection of explosive hazards at standoff distances, however, it is currently limited by low sensitivity^6^. Vibrational spectroscopies, on the other hand, promise to offer the required combination of chemical specificity and sensitivity. Vibrational spectra reflect the structure of molecules, providing endogenous and chemically specific signatures that can be exploited^7^.

Raman spectroscopy is a common experimental method to measure molecular vibrations: it is based on inelastic scattering of photons by a target molecule, returning scattered light with a frequency shift corresponding to the energy of vibrational modes of the molecule^8–14^. In spontaneous Raman (SR) a monochromatic beam at frequency $\omega _{pu}$ interacts with thermal molecular vibrations of frequency $\Omega$, giving rise to inelastically scattered (Stokes) light at frequency $\omega _{S}$ = $\omega _{pu}$ − $\Omega$. Due to the spontaneous nature of the process, the SR light is weak, spatially incoherent, and emitted in all directions, thus making stand-off detection very challenging^15,16^. This limitation can be overcome by coherent Raman scattering (CRS), a class of third-order nonlinear spectroscopy techniques which employ a sequence of light pulses to set up and detect a vibrational coherence within the ensemble of molecules at the laser focus. CRS combines two pulses, the pump and the Stokes, at frequencies $\omega _{pu}$ and $\omega _{S}$, respectively, to drive collective oscillations of those molecules whose vibrational frequencies $\Omega$ = $\omega _{pu}$ − $\omega _{S}$ match the pump-Stokes frequency difference.

The most widely employed CRS technique is coherent anti-Stokes Raman scattering (CARS)^17–19^, in which the vibrational coherence is read by a further interaction with a probe pulse at frequency $\omega _{pr}$, generating coherent radiation at the anti-Stokes frequency $\omega _{aS}$ = $\omega _{pr}$ + $\Omega$. According to this, the relevant anti-Stokes signal is inherently separated from the probe in the frequency domain, and it can be effectively detected by spectral filtering, which is an important advantage of CARS. On the other hand, CARS spectroscopy suffers from the so-called non-resonant background (NRB), which is a signal generated by the four-wave-mixing between pump, Stokes and probe, mediated by the non-resonant third-order nonlinear susceptibility of the target molecules and of the surrounding medium. The NRB is often much stronger than the resonant signal and, besides providing a large broadband plateau, it can severely distort the vibrational lineshapes.

**Figure 1:**
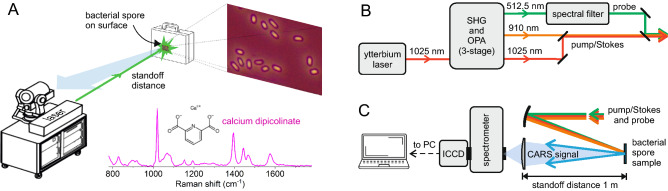
(**A**) Standoff detection of bacterial spores by a portable laser system; the inset shows the spontaneous Raman spectrum of calcium dipicolinate, which is the Raman marker for detection of bacterial spores. Layout of the system for (**B**) generation of pump, Stokes, and probe laser pulses and (**C**) excitation/collection of backscattering CARS signal in standoff configuration.

As the NRB relies only on non-resonant interactions with virtual states, it requires temporal overlap among the pump, Stokes and probe pulses, and can be greatly reduced or even completely suppressed in the so-called time-delayed CARS (TD-CARS)^20,21^, in which the probe pulse is delayed with respect to the pump/Stokes. A version of TD-CARS known as femtosecond adaptive spectroscopic technique via CARS (FAST-CARS) has been successfully applied to the detection of biological agents, such as endospores of the bacterium *Bacillus subtilis*^22,23^, a simulant for the harmful *Bacillus anthracis*, using the Raman signature of the molecule calcium dipicolinate (CaDPA) which is characteristic of spores. However, previous experiments relied on complex and bulky laser systems, based on Ti:sapphire technology, which are suitable for a laboratory environment but do not lend themselves to deployment in the field^22–30^. Recently, our group has demonstrated a setup for standoff detection of liquid chemicals by TD-CARS using a Yb-fiber laser and a hollow-core fiber for the generation of broadband pulses acting as the pump and Stokes at the same time in an impulsive stimulated Raman scattering configuration^31^. Using this setup, the excitation energy was primarily routed to the low-frequency portion of the Raman spectrum, while only a minor part was effectively used to excite and detect Raman modes at frequencies higher than 1000 cm^−1^, which prevented the application to more challenging samples such as powders, or biological materials. For this reason, despite promising results, standoff detection of bioagents based on CARS spectroscopy has not yet translated to real-world applications.

In this paper, we make a step forward in this direction by designing a broadband TD-CARS setup around a rugged, industrial grade ytterbium laser system, which offers compactness, portability and energy/power scalability, and applying it to the standoff detection of bacterial spores (Fig. 1A). We demonstrate detection of *Bacillus atrophaeus* spores at the standoff distance of 1 m, through its characteristic molecule CaDPA, with high signal-to-noise ratio (SNR) at a concentration of 10^5^ cfu/mm^2^ in an acquisition time of 1 s. Our system combines the sensitivity and specificity of CARS spectroscopy with improved portability, paving the way to a new generation of standoff detection systems for biological and chemical agents.

## Experiment and results

The experimental setup used for standoff detection is reported in Fig. 1B. It starts with an amplified Yb:KGW laser (Pharos, Light Conversion), generating 250-μJ, 250-fs pulses at 1025 nm (fundamental wavelength) and 1-kHz repetition rate. Three separate branches are derived from the Yb laser and used as the pump, Stokes and probe for CARS interaction. The first branch is the output of an optical parametric amplifier (OPA), pumped by the second harmonic (SH) of the Yb laser and seeded by a white light continuum (WLC); it provides 12-μJ (12-mW) pump pulses at 910 nm, with 35-nm (420-cm^−1^) full width at half maximum (FWHM) bandwidth, compressed to 30-fs duration by a prism pair. The second branch, with 90-μJ (90-mW) pulse energy at 1025 nm, is the residual of the fundamental beam after SH generation and gives the Stokes pulses with $\sim$7-nm (70-cm^−1^) FWHM bandwidth. Finally, the third branch starts with 120-μJ energy at 512.5 nm of the SH left unconverted after OPA interaction and spectrally narrows it in a zero dispersion pulse shaper, to provide 9-μJ (9-mW) probe pulses with $\sim$0.2-nm (8-cm^−1^) FWHM bandwidth. A detailed description and characterization of the OPA laser source can be found elsewhere^32^.

**Figure 2:**
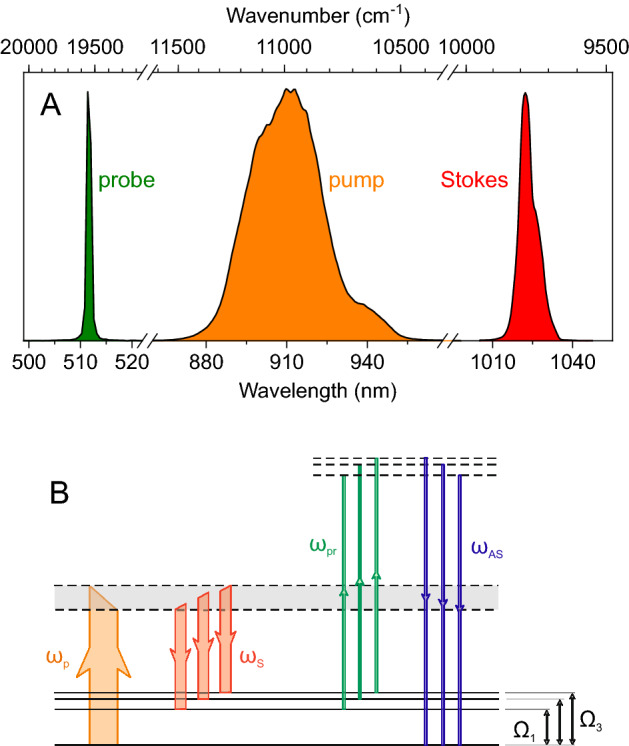
(**A**) The spectra of pump, Stokes, and probe pulses used for generation of CARS signal. The central frequency and bandwidth of pump and Stokes determine the range of Raman frequencies excited in the target; the bandwidth of the probe sets the final resolution of CARS spectra. (**B**) Scheme of the energy levels and transitions involved in broadband CARS detection (solid or dotted energy levels refers to actual or virtual levels, respectively). P: pump; S: Stokes; pr: probe; AS: anti-Stokes.

The spectra of the pump, Stokes and probe pulses are shown in Fig. 2A. The center wavelength of the pump pulse can be tuned by acting on the delay between the SH pulse and the WLC seed within the OPA, however, for the present application, it has been set to 910 nm to provide a frequency difference of 1250 cm^−1^ with respect to the Stokes pulse at 1025 nm. Taking into account the $\sim$500-cm$^{-1}$ convoluted bandwidth of the pump and Stokes pulses, this allows for efficient excitation of modes in the range 1000–1500 cm^−1^, thus covering a wide portion of the fingerprint region required for molecular characterization. The pump, Stokes and probe pulses are synchronized by delay lines and collinearly combined by dichroic beam splitters. They are then focused by a spherical mirror onto the target sample, placed at a standoff distance of 1 m (see Fig. 1C). The $1/e^2$ diameter of the three laser beams onto the target is 360 μm. The back-scattered CARS signal is collected by a 2-in. lens with 150-mm focal length and focused onto the entrance slit of a spectrometer equipped with an intensified CCD camera. The laser system presented here (Yb-laser and OPA) has a volume of nearly 1 m × 1.5 m × 0.3 m, a weight of 80 Kg, and a power consumption of $\sim$1.6 kW. The footprint can be largely reduced using OPA modules integrated to commercial mJ-class Yb lasers.

Figure  2B reports a scheme of the energy levels involved in the CARS signal generation. Broadband synchronized pump and Stokes pulses interact with virtual levels to create a vibrational coherence in the ensemble of molecules in the laser focus, simultaneously exciting a number of vibrations. This coherence is then read by the narrowband probe pulse, at frequency $\omega _{pr}$, which generates the spectrum at the anti-Stokes frequencies $\omega _{aS}=\omega _{pr}+\Omega _{i}$. In TD-CARS the probe pulse does not need to be synchronized with pump and Stokes, but can come at a delay $\tau$ comparable with the decay time of the vibrational coherence; in this case, the intensity of the CARS signal at a frequency $\Omega _{i}$ scales as $e^{-2\tau /T_{2vi}}$, where $T_{2vi}$ is the vibrational dephasing time of the mode at $\Omega _{i}$. As previously discussed, the CARS signal is superimposed on the NRB, contributing a broad and featureless spectrum which overlaps with the CARS peaks and can even distort the lineshapes, when it is very intense. The NRB requires temporal overlap of the pump, Stokes, and probe pulses, therefore, it can be strongly reduced by delaying the probe with respect to the pump/Stokes. By adjusting the delay of the probe pulse, one can thus optimize the detected CARS signal in order to reach a compromise between intensity and visibility of the vibrational peaks.

*Bacillus* spores are rich in CaDPA, a molecule representing around 15–20$\%$ of the dry spore mass^33^, depending on the species of interest. According to this, the Raman spectra of endospores are dominated by the vibrational modes of CaDPA at 659, 824, 1017, 1395, 1446, and 1572 cm$^{-1}$^34,35^ (see Fig. 1A). For standoff measurements, we first optimized the system using a sample of compressed sodium dipicolinate (NaDPA) powder, which is a substitute of CaDPA with almost the same Raman spectrum but easier to synthesize. After this, we switched to a sample of *B. atrophaeus* spores deposited onto the surface of a fused silica plate.

### Standoff detection of NaDPA

**Figure 3:**
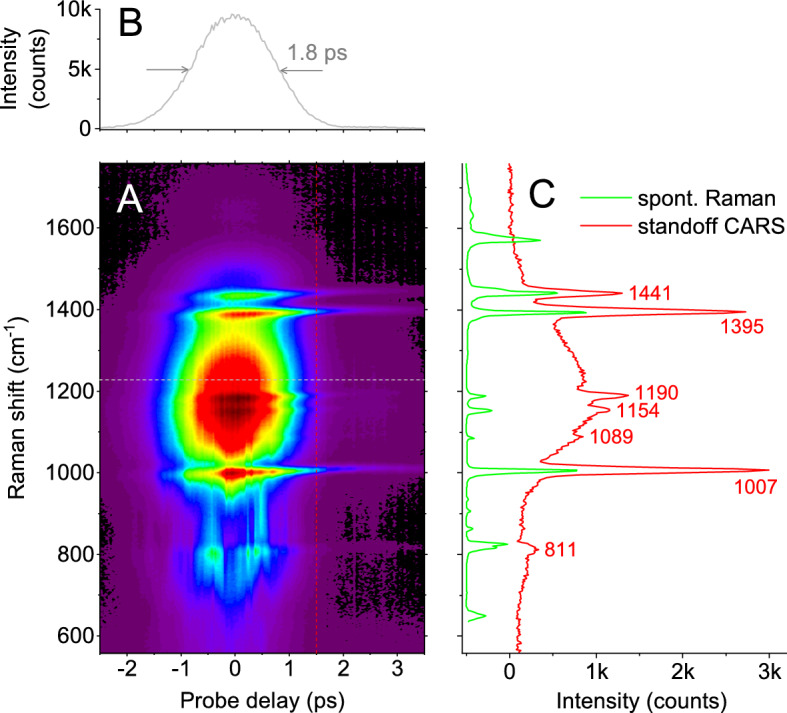
(**A**) Standoff CARS spectrogram of NaDPA powder. Pump, Stokes, and probe pulse energies are 4, 4, and 3 μJ, respectively. The spore sample is placed at 1 m from the laser system and the integration time is 100 ms. (**B**) Horizontal cross-section (gray line) at Raman shift of 1220 cm$^{-1}$ provides a measurement of probe pulse duration. (**C**) Vertical cross-section (red line) at probe delay of 1.5 ps provides a broadband standoff CARS spectrum of NaDPA; Raman spectrum (green line) of NaDPA powder acquired with a micro-Raman spectrometer^36^.

Figure 3A shows the CARS spectrogram, as a function of frequency and probe delay, of NaDPA powder as measured at a standoff distance of 1 m with an integration time of 100 ms. The energies of the pump, Stokes and probe pulses are 4, 4, and 3 μJ, respectively. At a probe delay of 1.5 ps, the NRB is significantly attenuated and almost all of the characteristic Raman peaks of NaDPA in the fingerprint region are resolved. The frequency axis has been referenced to the measured Raman peaks at 786, 1004, and 1210 cm$^{-1}$ (three-points linear fit) of neat toluene; the peak at 1004 cm$^{-1}$ has a FWHM of 8 cm$^{-1}$, which provides a measurement of the attainable resolution of CARS spectra. The accuracy on the determination of vibrational frequencies of NaDPA powder is ±2 cm$^{-1}$ as compared to data from a micro-Raman spectrometer^36^, confirming that distortion and frequency-shifting effects of CARS features due to interference with the NRB are negligible at probe delays of 1.5 ps. Interestingly, a horizontal cross-section of the spectrogram at 1220 cm$^{-1}$ (see Fig. 3B), a region where no molecular vibrations are present, provides a measurement of the convolution between the pump/Stokes and probe pulses, with a FWHM duration of 1.8 ps; taking into account the durations of pump and Stokes pulses (30 and 250 fs, respectively), the resulting duration of the probe pulse is $\sim 1.5$ ps, corresponding to a FWHM bandwidth of 0.25 nm (9.5 cm$^{-1}$), assuming a Gaussian profile of the probe spectrum, in agreement with the resolution observed in the CARS spectra. Table 1 shows the vibrational frequencies of NaDPA calculated from CARS measurements and corresponding assignments.

The amount of NaDPA powder contributing to the CARS signal shown in Fig. 3C is estimated to be $\sim$1 μg based on the sample density of 0.73 g/cm^3^, the laser beam diameter of 300 μm on the sample, and assuming an effective laser penetration depth of 10 μm^37^. Under these conditions, the SNR observed on the main Raman peaks at 1007 and 1395 cm$^{-1}$ is $\sim$280. As the concentration scaling law for SNR of backscattering CARS signal has been shown to be quadratic ($\propto N^2$, *N* being the number of molecules) also for pressed powder samples^38^, it can be inferred that a reduction of the amount of NaDPA to 100 ng, that is a factor of 10, would lead to a SNR of $\sim 3$, or a condition where the Raman signature of NaDPA is barely visible. However, the SNR has a square-root dependence on the integration time of the CCD camera sensor ($\propto \sqrt{n}$, *n* being the number of accumulated spectra), and can be improved by increasing the integration time from 100 ms to 1 s; this would restore the SNR to $\sim 10$, i.e. sufficiently good for discrimination of NaDPA Raman signature against the background. This estimate is consistent with the standoff CARS results obtained with bacterial spores, shown in the next section.

**Table 1:** Vibrational frequencies of NaDPA.

Assignment	CARS frequency (cm$^{-1}$)
C–H out-of-plane bend	811
C–C ring bend	1007
C–H bend	1089
C–H bend	1154
C–C ring stretch	1190
C–O stretch	1395
C–C ring stretch	1441

The second column lists the absolute frequencies calculated from CARS measurements in Fig. 3; the first column are the corresponding assignments^34–36^.

**Figure 4:**
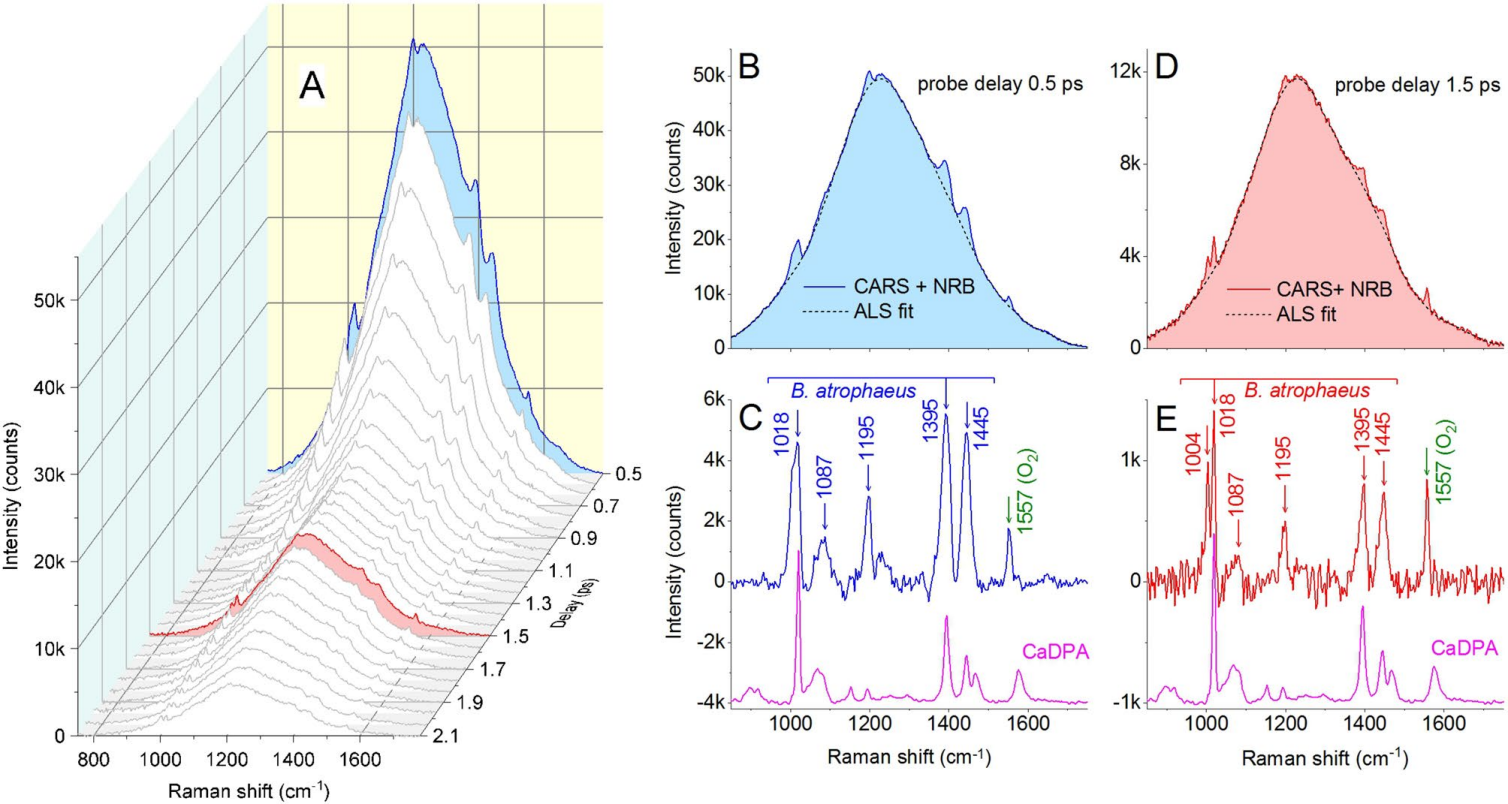
(**A**) Standoff CARS spectrogram of *B. atrophaeus* spores. Pump, Stokes, and probe pulse energies are 12, 12, and 9 μJ, respectively. The spore sample is placed at 1 m from the laser system and the integration time is 1 s. (**B**,**D**) Cross-sections (solid lines) of standoff CARS spectrogram of *B. atrophaeus* spores and ALS fitting profiles (dashed lines) for probe delays of 0.5 and 1.5 ps. (**C**,**E**) Corresponding residuals and reference Raman spectrum of CaDPA (magenta line). Residuals represent broadband CARS fingerprints of *B. atrophaeus* spores.

### Standoff detection of *B. atrophaeus* spores

Standoff CARS spectra of *B. atrophaeus* spores are shown in Fig. 4. The spore sample is placed again at a distance of 1 m from the laser system but the integration time is now increased to 1 s. Figure 4A shows the spectra corresponding to probe delays from 0.5 to 2.1 ps, that is the window in which the CARS signature of spores is best observed. The resonant contribution is retrieved by fitting the NRB (asymmetric least square fit, ALS^39^) and subtracting the fit from the raw spectra. Figure 4B,D show the spectra acquired at 0.5 and 1.5 ps, respectively, along with their ALS fit; the retrieved resonant CARS spectra of spores are shown in Fig. 4C,E.

At 0.5 ps, the pump, Stokes, and probe pulses are well overlapped and the intensities of spore Raman resonances are relatively strong, especially those at 1017, 1395, and 1445 cm$^{-1}$, where a SNR larger than 80 is observed. This triplet provides a unique combination of frequencies which can be confidently ascribed to CaDPA^40,41^ and, because of the high SNR, can be readily used for classification of the marker by an automated software routine. It should be noted that the resonant contribution here is distorted by the interference with the NRB and the Raman peaks appear less resolved.

Higher spectral quality and resolution are obtained for a probe pulse delay of 1.5 ps. The interference with the NRB is reduced in this case, as shown by the measurements on NaDPA, and the resolution is ultimately limited by the 8-cm$^{-1}$ bandwidth of the probe pulse. This is confirmed by the CARS spectrum in Fig. 4E, where the doublet of lines at 1004 and 1018 cm$^{-1}$ is well resolved; this doublet is specific to bacterial spores, as the line at 1004 cm$^{-1}$ is due to phenylalanine in spore protein and that at 1017 cm$^{-1}$ to the “breathing” mode of pyridine in CaDPA molecule^41^. The spectral features at 1087 cm$^{-1}$ (pyridyne: trigonal ring breathing, or DNA:O-P-O$^-$ stretching)^34,42^ and 1195 cm$^{-1}$ (CaDPA: CH bend)^36^ provide additional details for recognition of the Raman fingerprint of spores. The sharp Raman line at 1557 cm$^{-1}$ is due to atmospheric oxygen (O$_2$). At this delay, however, the amplitude of the Raman signal is decreased by a factor of $\sim$4 (compare Fig. 4C,E), due on the one hand to vibrational dephasing and, on the other hand, to the reduction of the heterodyne amplification effect by the NRB. One should therefore carefully optimize the probe delay trading spectral intensity for frequency resolution.

Similar to NaDPA powder, the amount of CaDPA in the spores contributing to the CARS signal can also be estimated. On average, a single spore of *B. atrophaeus* contains 49 fg of CaDPA salt in the core, because the mass of a dry spore is 328 fg^43^, and the relative mass of CaDPA is generally assumed to be around 15$\%$. As the mean volume of *B. atrophaeus* spores is expected to be of 0.27 μm^3^^43^, we assumed an effective volume of 1 μm^3^ for the close packed spores of our deposited sample; according to this, the number of spores contained within the volume probed by the laser beam ($1.4\times 10^6$ μm^3^) is estimated to be $1.4\times 10^6$, corresponding to 70 ng of CaDPA contributing to the CARS signal. According to Fig. 4E, this provides a SNR of 20 on the main peak at 1018 cm$^{-1}$, which is in close agreement with the data extrapolated from NaDPA results.

## Discussion

Real-world applications for standoff detection of biological warfare agents require the detection of bacterial spores with high sensitivity. To this purpose, we improved the signal intensity by an optimized combination of the pulse wavelengths in our CARS system. On the one hand, we used near-IR wavelengths of 920/1030 nm for the pump/Stokes preparation pulses, which allows for higher energies (fluences) without destroying the spore sample, especially in the presence of intense 30-fs pump pulses, and hence results in stronger CARS signal. On the other hand, the choice of visible probe pulses at 512.5 nm provides improved scattering efficiency with respect to the near-IR, according to $1/\lambda ^4$ Rayleigh scattering law, and, in addition, shifts the CARS signal in the range 480–490 nm, where the intensified CCD camera has the highest efficiency. Besides signal intensity, broadband operation is also important for improving the specificity, as the observation of several spectral features improves recognition of Raman signatures. The preparation pulses that we used in our system provide a combined band of 500 cm$^{-1}$, which addresses most of the fingerprint region of bacterial spores, and enables confident detection of their marker.

For operation at standoff distances, a large depth of focus is needed in order to reduce the sensitivity to variations of target distance. Our design relaxes the requirements on target position by using a relatively large beam diameter of 360 μm for all three beams onto the target, corresponding to a Rayleigh range of $\sim$0.1 m for the Stokes beam at 1025 nm, for which the divergence is larger. Assuming the depth of focus is twice the Rayleigh range of the Stokes beam, and considering a target distance of 1 m, the resulting tolerance to target position is ±10$\%$, which is largely acceptable for a standoff CARS system where the signal intensity is proportional to the product of intensities of the pump, Stokes, and probe according to the law $I_{CARS}\propto I_{pu}\times I_{S} \times I_{pr}$. The previous state-of-art system for bacterial spore detection using FAST-CARS spectroscopy was based on strong focusing of the lasers down to diameters of 40 μm and target distance of 10 cm, with a resulting tolerance to target position of ±1%^23^.

In addition, the adoption of relatively large beam diameters onto the sample allows for increased pulse energy while keeping fluence and peak intensity well below the damage threshold, resulting in a higher CARS energy collected by the spectrometer. More specifically, the standoff CARS spectra shown in Fig. 4 were measured using pump pulse energy of 12 μJ, corresponding to a fluence of 0.024 J/cm^2^ and an intensity of 0.7 × 10^12^ W/cm^2^ at a pulse duration of 30 fs, that is well below the reported spore damage threshold of 0.2 J/cm^2^ and 3 × 10^12^ W/cm^2^ for fluence and intensity, respectively^23^. We note that the CARS radiation diffused by the spore sample is collected by a 10-cm focal length lens at 1-m distance, and the resulting diameter of the CARS beam imaged onto the spectrometer input is 39 μm, which can be almost entirely accepted by the spectrometer input slit to achieve the spectral resolution of 0.2 nm.

## Conclusion

Optical techniques offer a great promise for rapid standoff detection of biological threats, to enable the user to operate at a safe distance from the hazard. CARS spectroscopy, in particular, combines the molecular specificity of Raman spectroscopy with the high signal levels afforded by a coherent technique and has already demonstrated the capability to classify bacterial spores. However, for real-world application of this technique, improvements are needed in sensitivity and systems need to be designed for deployment in the field. In our work we designed the optical system for the detection of bacterial spores starting from a compact and rugged industrial grade Yb laser, which is a step towards translating from the laboratory to operating in more real-world environments. We have further selected the wavelengths of the pump/Stokes/probe beams in order to optimize the process and maximize the intensity of the detected CARS signal while preventing photodamage of the spores. Using this system we have been able to detect, at a standoff distance of 1 m, *B. atrophaeus* spores at a concentration of 10^5^ cfu/mm^2^, with a signal to noise ratio of 20 and an acquisition time of 1 s.

Our experiments have been so far performed at moderate output energies of 250 μJ from the driving laser. Using a similar all-solid-state Yb technology, the output energy can be readily increased by more than an order of magnitude, allowing to increase the generated CARS intensity by nearly three orders of magnitude before reaching the damage threshold of the spores. Further scaling of the generated CARS pulse energy can be achieved by increasing the beam diameters at the sample. Considering the $\propto 1/d^2$ scaling of the CARS signal with standoff distance d, we can anticipate an increase in detection distance by over an order of magnitude, to the tens of meters range. An additional improvement in sensitivity can be achieved using machine learning (ML) algorithms to process the spectra. ML can classify specific chemical fingerprints in the presence of interfering factors, such as stray environmental light, or inherent variations of the properties of the sample and the substrate. Our standoff CARS system can be further extended to the detection of chemical warfare agents or, more generally, to environmental sensing.

## Methods

### Chemicals

Dipicolinic acid (DPA, CAS n. 499-83-2) was obtained from Sigma Aldrich. Sodium dipicolinate (NaDPA) was prepared from DPA and a stoichiometric amount of sodium hydroxide (NaOH, CAS n. 1310-73-2), diluted in distilled water prior to mixing with DPA powder. A 250-mM NaOH/DPA water solution has been evaporated for a few hours at 30 °C, and then the NaDPA crystals formed at the walls of the container have been crushed into a fine powder, loaded into a cylindrical press die, and pressed by a 10-ton hydraulic press in order to get the solid sample used for standoff CARS experiment. The density of the pressed NaDPA sample is $\sim$0.73 g/cm^3^, as calculated from the weight of 240 mg, diameter of 12.70 mm, thickness of 1.30 mm.

### *Bacillus atrophaeus* strains and spore preparation

A seed stock of spores was inoculated on to Tryptone Soya Agar (TSA) plates and incubated overnight at 37 °C. Individual colonies were picked into 10 ml L-broth and incubated overnight at 37 °C. This starter culture was used to inoculate larger flasks of Leighton Doi sporulation media (LD), 500 ml flasks containing 200 ml LD per flask. These were incubated at 30 °C at 160 rpm rotation for 7 days (to culture the vegetative cells, then provide time for the cells to sporulate). The spores were pelleted by centrifugation at 8000 rpm at 4 °C for 10 minutes, then resuspended in distilled water. The spores were then washed three times by centrifugation and resuspension in distilled water as above. The final spore preparation was resuspended in distilled water to the required concentration and stored at 4 °C. The spore concentration was calculated by plating dilutions on to TSA plates, incubating them at 37 °C overnight and then counting the colonies. An aliquot was analysed for spores by 100× magnification oil immersion phase-contrast microscopy, and showed very few vegetative cells and mostly spores.

## Data Availability

The datasets used and analyzed in this study are available from the corresponding author upon reasonable request.
